# Exogenous Cushing Syndrome and Hip Fracture Due to Over-the-Counter Supplement (Artri King)

**DOI:** 10.7759/cureus.41278

**Published:** 2023-07-02

**Authors:** Suhail M Saad-Omer, Mustafa Kinaan, Moises Matos, Hanford Yau

**Affiliations:** 1 Department of Internal Medicine, University of Central Florida College of Medicine, University of Central Florida/HCA Florida Healthcare Graduate Medical Education, Orlando, USA; 2 Department of Internal Medicine, University of Central Florida College of Medicine, Orlando Veterans Affairs Medical Center, Orlando, USA

**Keywords:** hip fracture, exogenous, cushing syndrome, osteoporosis, over-the-counter supplement, hypercortisolism, arti king, cushing’s

## Abstract

The most common cause of Cushing syndrome (CS) is exposure to exogenous glucocorticoids. There is an increasing incidence of adulterated over-the-counter (OTC) supplements containing steroids. We present a case of Artri King (AK)-induced CS in a 40-year-old woman who presented with an intertrochanteric fracture of her right femur. Laboratory testing revealed suppressed cortisol and adrenocorticotropic hormone, which was consistent with suppression of the hypothalamic-pituitary-adrenal (HPA) axis. Following the cessation of the AK supplement, the patient’s HPA axis recovered, and the clinical manifestations of CS improved. This case emphasizes the need for better regulation of OTC supplements and the need for cautious use.

## Introduction

Cushing syndrome (CS) is a condition that occurs because of high blood levels of glucocorticoids (GCs). These patients can present with a variety of systemic signs and symptoms, including truncal obesity, easy bruising of the skin, violaceous abdominal striae, resistant hypertension, dysglycemia, as well as osteoporosis. CS can occur because of adrenal etiologies such as adrenal adenoma, adrenal cancer, or adrenal hyperplasia or from an adrenocorticotropic hormone (ACTH)-producing pituitary adenoma or ectopic tumor. However, the most common cause of CS is the exogenous administration of GCs [1]. While exogenous GCs are often medically prescribed for the treatment of inflammatory conditions, some patients may be accidentally exposed to exogenous GCs from over-the-counter (OTC) supplements. We present a case of a young woman who developed exogenous CS and suffered a hip fracture as a result of taking an OTC supplement, Artri King (AK), adulterated with GCs.

## Case presentation

**Figure 1:**
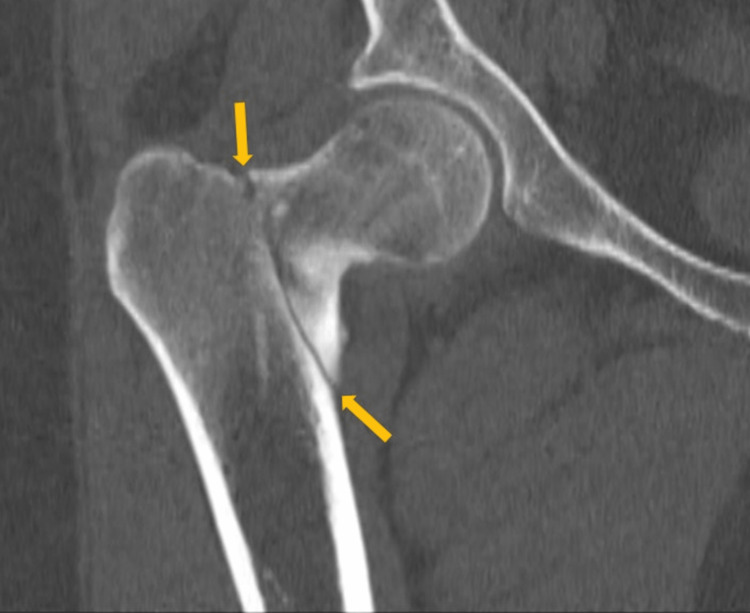
A CT scan showing the right intertrochanteric fracture of the right femur (yellow arrows)

A 40-year-old obese woman presented to the hospital following a fall at home. She reported a snapping noise and sudden right hip pain while trying to stand up, and subsequently fell to the floor. She had noted right-sided hip pain for several days preceding her fall. She was evaluated in the emergency department where computed tomography (CT) imaging of the right lower extremity showed an intertrochanteric fracture of the right femur (Figure 1). The patient underwent open reduction and internal fixation of her right femur. The patient reported an unexplained weight gain of approximately 40 lbs in the preceding five months with a peak weight of 223 lbs (101 kg) and a body mass index (BMI) of 37 kg/m2. The patient denied taking any medications or supplements at the time of hospitalization. The endocrinology team was consulted to evaluate for causes of secondary osteoporosis in this young woman.

Diagnostic assessment

Her vital signs showed a blood pressure of 142/96 mmHg, heart rate of 68 beats per minute, temperature of 98.1°F (36.7°C), and 98% oxygenation on room air. Physical examination did not reveal abdominal striae or buffalo hump. She did have supraclavicular fat deposition and central obesity. No proximal muscle weakness was present.

**Figure 2:**
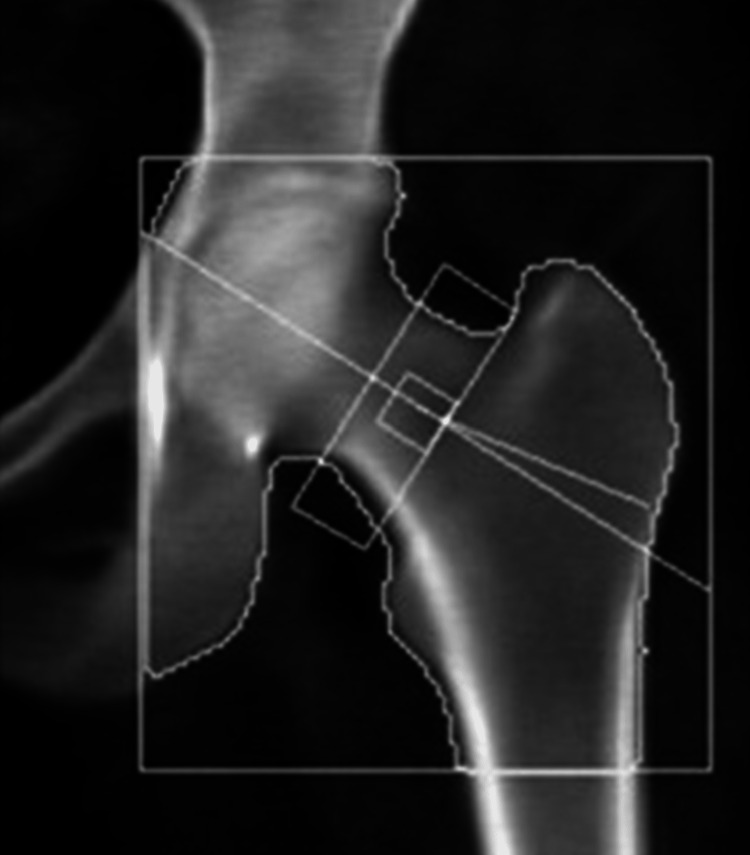
Dual-energy X-ray absorptiometry (DEXA) scan of the femoral neck showing osteopenia

**Figure 3:**
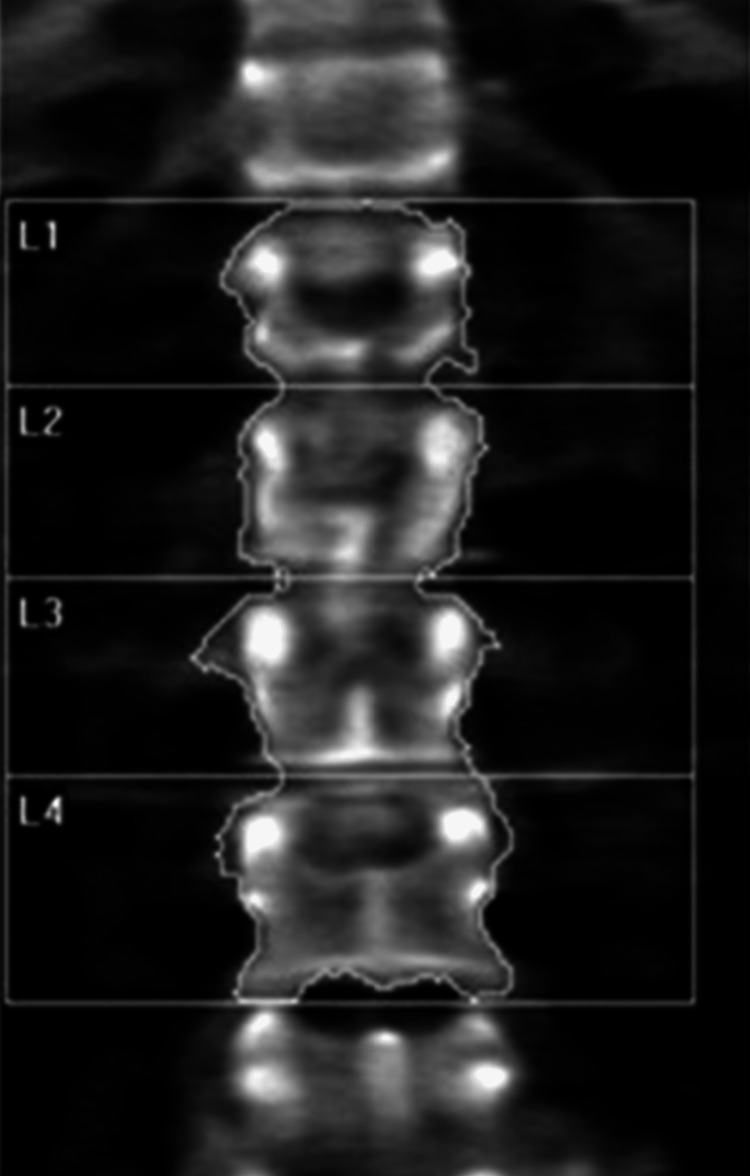
Dual-energy X-ray absorptiometry (DEXA) scan of the lumbar spine showing osteoporosis

Laboratory tests were pertinent for decreased 25-hydroxy vitamin D, increased parathyroid hormone (PTH), and normal calcium (Table 1). These findings were consistent with secondary hyperparathyroidism due to vitamin D deficiency. Dual-energy X-ray absorptiometry (DEXA) scan revealed osteoporosis (Figures 2, 3 and Tables 2, 3). Further testing showed normal thyroid-stimulating hormone (TSH), estradiol, follicle-stimulating hormone (FSH), and luteinizing hormone (LH), thus ruling out hyperthyroidism and primary ovarian insufficiency as possible causes of reduced bone mineral density (Table 1). Random cortisol was checked as hypercortisolism was suspected but it was found to be decreased along with decreased ACTH as well (Table 4). A cosyntropin stimulation test was performed, which showed decreased baseline cortisol with inappropriately decreased cortisol levels at 30 minutes and 60 minutes (Table 5). Given the discordance between the patient’s presentation and the lab results, assay interference was suspected, and further evaluation of the adrenal function was performed. Repeat labs using liquid chromatography-mass spectrometry (LCMS) assay again confirmed persistently low cortisol (Table 4). A 24-hour free urine cortisol was too low to quantify per assay despite the adequate volume. Further evaluation showed overall low adrenal steroids, including deoxycorticosterone, 17-hydroxyprogesterone, androstenedione, 11-deoxycortisol, pregnenolone, dehydroepiandrosterone sulfate, corticosterone, and progesterone.

**Table 1:** Patient's lab values on admission

Lab test	Patient's value	Reference range
25-hydroxy vitamin D	12.8 ng/ml	30-100 ng/ml
Parathyroid hormone (PTH)	86.2 pg/ml	10-66 pg/ml
Serum calcium	9.5 ng/dl	8.8-10.5 mg/dl
Thyroid-stimulating hormone (TSH)	2.49 mIU/L	0.36-3.74 mIU/L
Estradiol	57.1 pg/ml	19.8-144.2 pg/ml
Follicle-stimulating hormone (FSH)	5.4 mIU/ml	2.5-10.4 mIU/ml
Luteinizing hormone (LH)	6 mIU/ml	1.9-12.5 mIU/ml

**Table 2:** Summary of dual-energy X-ray absorptiometry (DEXA) scan results of the femoral neck

Region	Area (cm^2^)	Bone mineral content (g)	Bone mineral density (g/cm^2^)	T-score	Peak reference	Z-score	Age-matched
Femoral neck	4.76	3.53	0.742	-1.0	87	-0.7	91
Total	33.39	26.14	0.783	-1.3	83	-1.1	85

**Table 3:** Summary of dual-energy X-ray absorptiometry (DEXA) scan results of the lumbar spine

Region	Area (cm^2^)	Bone mineral content (g)	Bone mineral density (g/cm^2^)	T-score	Peak reference	Z-score	Age-matched
L1	10.79	7.56	0.701	-2.6	71	-2.4	73
L2	11.79	9.06	0.768	-2.4	75	-2.1	77
L3	12.70	9.98	0.786	-2.7	73	-2.4	75
L4	15.57	11.42	0.733	-3.0	69	-2.7	71
Total	50.86	38.03	0.748	-2.7	71	-2.5	73

**Table 4:** Patient's cortisol and adrenocorticotropic hormone levels before and after stopping Artri King

Lab test	Patient's values while on Artri King	Patient's values four weeks off of Artri King	Reference range
Random cortisol (routine assay)	<0.64 μg/dL	7.3 μg/dL	5-25 μg/dL
Adrenocorticotropic hormone (ACTH)	1.5 pg/ml	12 pg/ml	7.2-63.3 pg/ml
Random cortisol (using liquid chromatography-mass spectrometry (LCMS) assay)	0.526 μg/dL	N/A	5-25 μg/dL

**Table 5:** Results of cosyntropin test while on Artri King

Cosyntropin stimulation test	Patient value	Reference range
Baseline cortisol	1.64 μg/dL	5-25 μg/dL
Cortisol after 30 minutes	1.33 μg/dL	>18 μg/dL
Cortisol after 60 minutes	6.48 μg/dL	>18 μg/dL

Treatment

She was started on teriparatide as well as vitamin D and calcium supplementation for the treatment of osteoporosis. Based on the aforementioned testing and the apparent symptoms of hypercortisolism, the patient was questioned again about the potential intake of steroids. She then recalled that she had been taking AK, an OTC supplement promoted for joint pain and arthritis. She reported that she had been taking two tablets of the supplement three times a day intermittently for the past three years. The patient neglected to bring it to the medical team’s attention before because she was under the impression that it was a multivitamin and did not have implications on her diagnosis. She was asked to stop the supplement and was educated about potential adrenal insufficiency symptoms and GC withdrawal.

Outcome and follow up

Repeat labs after four weeks off AK showed improved cortisol and ACTH levels indicating recovery of her hypothalamic-pituitary-adrenal (HPA) axis (Table 4). She lost 25 lbs in this time span with lifestyle modification. She continues teriparatide for osteoporosis, and monitoring of her bone mineral density is planned.

## Discussion

This patient initially presented with a pathological fracture of her right femoral head. Given her young age, causes of secondary osteoporosis, including CS, were explored. The prevalence of osteoporosis in CS patients is 50% [2]. The effects of GC on bone health have been well studied. The major mechanism by which GC affects bone mineral density is by impairment of bone formation. GCs increase osteoblast and osteocyte apoptosis and decrease osteoblast function through their catabolic effects, which result in a dramatic decrease in bone formation rate. A prolonged lifespan of osteoclasts is observed with GC. A decrease in bone formation markers such as P1NP and osteocalcin has been observed in patients treated with GC [3]. Long-term GC use is associated with increased risk for fractures with a reported global prevalence of fractures of 30-50%. The risk for vertebral fractures is even higher, particularly in the thoracic and lumbar vertebrae. Interestingly, the risk for fracture with GC use peaks early in the course of treatment, often as early as three months into treatment, and declines rapidly after GC discontinuation [4]. An increased fracture risk has been described even with relatively low doses of GC (2.5-7.5 mg of prednisone or other equivalently dosed GC) and even with short-term use of under 30 days [5].

Our patient’s initial labs confirmed adrenal suppression despite our initial suspicion of CS, given her ongoing weight gain, central obesity, and osteoporosis. However, no obvious source of exogenous GC was identified. In most cases, the source of exogenous GC is easily identified through medication reconciliation; however, in our case, the patient was inadvertently exposed to steroids from an unregulated supplement, AK. The supplement’s ingredients were listed as glucosamine, chondroitin, collagen, vitamin C, curcumin, methylsulfonylmethane, nettle, and omega-3 fatty acids, with no mention of any steroid components. In a letter to the editor of the Internal Medicine magazine, several doctors published their concerns about a recent increase in CS cases associated with the use of AK and other similarly unregulated products [6]. Based on our literature search, three similar cases were published [7,8]. The reported cases developed CS after taking Artri King for several months, but none of them presented with a fracture.

A warning by the U.S. Food & Drug Administration (FDA) was issued on April 20, 2022, indicating that FDA laboratory testing of this supplement confirmed the presence of undeclared drug ingredients, including dexamethasone, methocarbamol, and diclofenac. The FDA, however, was unable to confirm the exact amount of dexamethasone that these supplements contained [9]. Adverse events, including liver toxicity and death, were reported by the FDA.

One study revealed that between 2007 and 2016, the FDA had issued more than 700 warnings about the sale of dietary supplements that contained unlisted and potentially dangerous ingredients. The majority of these supplements included those marketed for sexual enhancement, weight loss, or muscle building [10]. This case highlights the risks of undisclosed ingredients in OTC supplements.

## Conclusions

In conclusion, we recommend that a thorough reconciliation of medication and supplements be obtained for all patients with CS. Supplements should be stopped and HPA axis testing should be repeated in patients with suspected exogenous GC exposure, even if steroids are not declared in the ingredients. It is also important to monitor such patients for adrenal insufficiency due to GC withdrawal and consider GC tapering if necessary. Our patient showed improvement in cortisol levels with no overt symptoms of adrenal insufficiency without the need for GC therapy. This case demonstrates the first case of AK-induced CS resulting in a pathological fracture. Given the increased use and availability of OTC supplements, this case highlights on the importance of detailed history-taking and the role of supplements in causing CS. This case also stresses the need for further education and counseling of our patients as well as tighter control on the manufacturing and sale of these supplements.
